# Microstructure, Tensile, and Fatigue Properties of Large-Scale Austenitic Lightweight Steel

**DOI:** 10.3390/ma15248909

**Published:** 2022-12-13

**Authors:** Jong-Ho Shin, Jeon-Young Song, Sung-Dae Kim, Seong-Jun Park, Young-Wha Ma, Jong-Wook Lee

**Affiliations:** 1 Corporate Research and Development Institute, Doosan Enerbility Co., Ltd., 22 Doosanvolvo-Ro, Changwon 51711, Republic of Korea; 2 Department of Materials Science & Engineering, Pukyong National University, Busan 48513, Republic of Korea; 3 Department of Steels, Advanced Metals Division, Korea Institute of Materials Science, 797 Changwondae-Ro, Changwon 51508, Republic of Korea

**Keywords:** lightweight steel, κ-carbide, low cycle fatigue (LCF), steam turbine, blade

## Abstract

High-Mn lightweight steel, Fe-0.9C-29Mn-8Al, was manufactured using steelmaking, ingot-making, forging, and rolling processes. After the final rolling process, a typical austenite single phase was observed on all sides of the thick plate. The microstructural changes after annealing and aging heat-treatments were observed, using optical and transmission electron microscopy. The annealed coupon exhibited a typical austenite single phase, including annealing twins in several grains; the average grain size was 153 μm. After aging heat treatment, κ-carbide was observed within the grains and on the grain boundaries. Additionally, the effect of aging heat treatment on the mechanical properties was analyzed, using a tensile test. The fine κ-carbide that precipitated within the grains in the aged coupon improved the 0.2% offset yield and the tensile stresses, as compared to the as-annealed coupon. To estimate the applicability of high-Mn lightweight steel for low-pressure (LP) steam turbine blades, a low-cycle fatigue (LCF) test was carried out at room temperature. At a total strain amplitude of 0.5 to 1.2%, the LCF life of high-Mn lightweight steel was approximately three times that of 12% Cr steel, which is used in commercial LP steam turbine blades. The LCF behavior of high-Mn lightweight steel followed the Coffin–Manson equation. The LCF life enhancement in the high-Mn lightweight steel results from the planar dislocation gliding behavior.

## 1. Introduction

Lightweight steel has high strength and toughness, and its density is approximately 10% lower than that of conventional steels. Thus, it is being developed as a candidate material to improve energy efficiency and reduce CO_2_ emissions in automobiles [1,2,3]. Lightweight steel is classified into Fe-Al and Fe-Mn-Al-C-based steels. This study investigated the effect of aging heat treatment on the microstructural evolution and mechanical properties of Fe-Mn-Al-C-based steels, including high-Mn and C concentrations that were added to stabilize the austenite structure at room temperature [4,5]. Fe-Mn-Al-C-based steels are precipitation-hardened by κ-carbide ((Fe,Mn)_3_AlC) with a perovskite E21 structure within the grains during aging heat treatment. However, when the steel is aged at high temperatures for an extended period, coarse κ-carbide precipitates on the grain boundaries, reducing the toughness of the steel [5]. For steels containing high concentrations of Mn and Al, β-Mn is also precipitated on the grain boundaries during aging heat treatment at a temperature of 550 °C or higher, thereby reducing the toughness of the alloy [6].

As compared to the conventional 12% Cr steel used for steam turbine blades, the investigated steel has a lower material cost and lower density, which can reduce centrifugal force. A lower centrifugal force on the blades can allow for longer blades, improving the efficiency of the power plant. Thus, it can be considered for use as a blade material for steam turbines rotating at high speed. Steam turbines consist of high-pressure (HP), intermediate-pressure (IP), and low-pressure (LP) turbines. Here, the temperature of the steam injected into the HP and IP turbines is approximately 600 °C, whereas the temperature of steam injected into LP turbines is approximately 350 °C. Since κ-carbide precipitates at temperatures exceeding 400 °C, it is presumed that the steel is suitable for use in LP turbine blades [5,7]. LP turbines, as compared to the HP and IP turbine blades, are damaged because of fatigue, stress-corrosion cracking, and corrosion fatigue [8,9]. However, they are not damaged by creep, because LP turbine blades operate below 350 °C. The blades undergo repeated loading by the start-up and shut-down cycles of steam turbines; therefore, a low-cycle fatigue (LCF) life is an important criterion for blade design [10].

The tensile deformation mechanisms, which are precipitate types according to the heat-treatment conditions and mechanical properties of the precipitates of Fe-Mn-Al-C-based steels, have been studied using steel manufactured with small-sized ingots [1,11,12,13]. However, there are few cases where large-scale lightweight steel was manufactured using steelmaking, forging, and rolling processes in facilities that can produce large-scale steels. Additionally, literature on the tensile and fatigue properties of large-scale lightweight steels is limited.

Therefore, the purposes of this paper are to observe the microstructural characteristics of Fe-Mn-Al-C lightweight steel and to evaluate the tensile properties of large-scale lightweight steel manufactured by forging and rolling processes using 4-ton class ingots. Additionally, the LCF properties of the large-scale lightweight steel were evaluated to consider the applicability of Fe-Mn-Al-C lightweight steel to LP turbine blades and compared to the LCF properties of the conventional 12% Cr steel currently used for LP turbine blades.

## 2. Experimental Procedure

**Figure 1:**
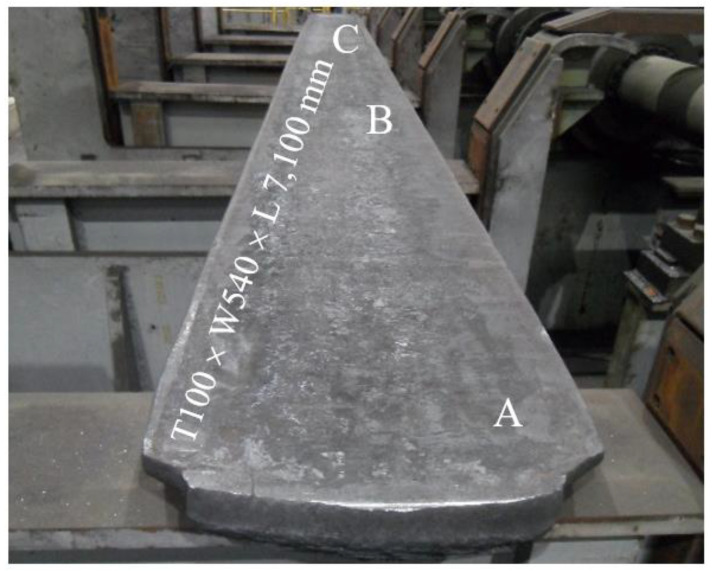
Appearance and dimensions of the as-rolled lightweight steel; A, B, and C represented the locations where the microstructural samples were taken form the as-rolled plate.

Table 1 shows the chemical composition of the steel used in this study. After steelmaking in an electric arc furnace and degassing in a refining furnace, the molten steel was bottom-poured to produce a 4-ton class ingot at atmospheric pressure. The 4-ton class ingot was forged to an approximately 260 mm × 510 mm × 2850 mm square bar (thickness, width, and length, respectively) under the forging temperature range of 950 to 1200 °C. Then, the forged square bar was hot-rolled to 100 mm × 540 mm × 7100 mm (thickness, width, and length, respectively) at a rolling temperature range of 950 to 1180 °C (Figure 1). The density of the steel was 6.90 g/cm^3^, which was measured with a densimeter (SD-200L; Alfa mirage, Osaka-Shi, Japan) using Archimedes’ principle. The microstructure of the coupons obtained from the A, B, and C regions shown in Figure 1 were examined. The coupons were removed from the large-size plate using a water jet cutting machine and the coupons were then cut into smaller sizes using a band saw.

The rolled plate was subjected to annealing and aging heat treatment to determine the microstructure, tensile, and LCF properties at room temperature. To control the microstructure of the plate, the rolled plate was annealed at 1050 °C for 5 h, followed by water quenching, and it was then aged at 500 °C for 10 h, followed by air cooling.

Microstructural observations of the specimens obtained from the coupon before/after aging heat treatment were performed using optical microscopy (OM; ZEISS Axioplan, Germany) and transmission electron microscopy (TEM; JEOL JEM-2100F, Japan). The specimens were prepared for OM observation by mechanical polishing and chemical etching, using a nital solution. For the TEM observation, discs with diameters of 3 mm and thicknesses of 0.1 mm were electrochemically etched with a mixed solution of 10% Perchloric acid and 90% methanol at −30 °C, using a twin-jet electrolytic polishing machine (Struers, TenuPol-5, Denmark) at 20 V and 70 mA.

Tensile samples with diameters of 6.25 mm and gauge lengths of 25 mm were prepared according to ASTM A370 and a tensile test was performed using Instron 5982 at room temperature, according to ASTM E-8M. The LCF test specimen, obtained from the plate along the rolling direction, was prepared as a 6.35 mm-diameter cylinder with a gauge length of 18.5 mm. The LCF test was performed, according to ASTM E606, in the fully reversed axial strain-control mode with a triangular waveform. The strain rate and the strain ratio (R) were 0.001 s^−1^ and −1, respectively. The total strain amplitude (Δ*ε_t_*/2) was changed from 0.5 to 1.2% (MTS, Landmark). The fatigue life was defined as a 20% load drop point of a stabilized peak load. An LCF test was also carried out on commercial 12% Cr steel under the same LCF test conditions as those of the lightweight steel, for comparison.

## 3. Results and Discussion

### 3.1. Microstructure of Large As-Rolled Plate

**Figure 2:**
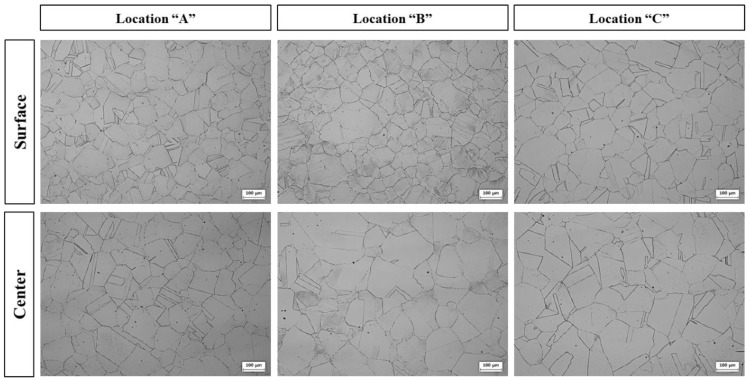
Optical micrograph showing the microstructures at each location of the as-rolled lightweight steel shown in Figure 1.

Figure 2 shows the microstructure at each location along the thickness direction of the rolled plate. After rolling, a typical austenite single phase, composed of annealing twins within several grains, was observed in all selected observation locations; the average grain size was approximately 102 μm. Additionally, no δ-ferrite was observed. It was assumed that dynamic recrystallization during the rolling process resulted in fine grains and uniform grain size.

As shown in Figure 2, non-metallic inclusions were not observed, and oxygen and nitrogen were measured as 4 ppm and 13 ppm, respectively. It was assumed that the Al added during the refining process combined with O and N to form Al_2_O_3_ and AlN in the molten metal, which were separated from the molten metal by flotation [14].

### 3.2. Microstructural Evolution and Tensile Properties after Aging Heat Treatment

**Figure 3:**
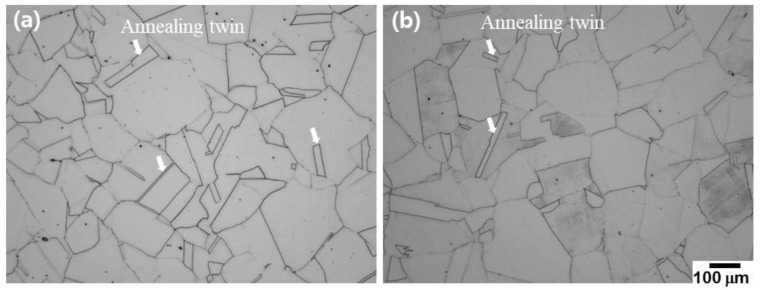
Optical micrograph showing the microstructures after (**a**) annealing heat treatments and (**b**) aging heat treatments.

Figure 3 shows OM micrographs of the large as-rolled plate after annealing heat treatment and aging heat treatment. After annealing heat treatment, a typical austenitic single phase and annealing twins within a few grains were observed. Additionally, the grain size was approximately 153 μm, which was coarsened by approximately 50 μm, compared with that of the as-rolled plate.

**Figure 4:**
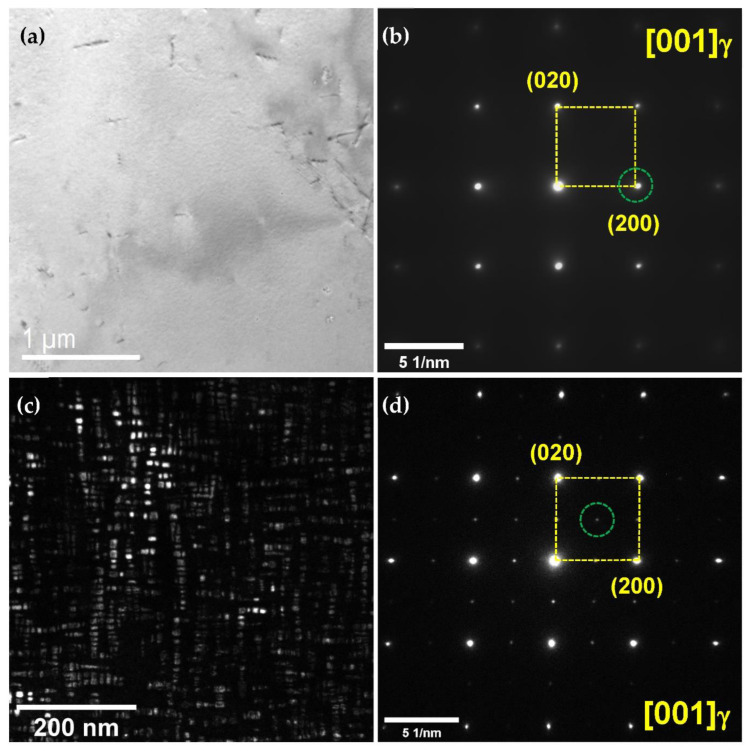
(**a**) Bright-field (BF) TEM image and (**b**) selected area diffraction pattern (SADP) of the as-annealed lightweight steel; (**c**) dark-field (DF) TEM image and (**d**) SADP of the aged lightweight steel. The DF-TEM image (**c**) was acquired using the diffraction spot of the κ-carbide, which is highlighted as a dotted green circle in (**d**).

To determine the effects of the aging heat treatment on the microstructure, TEM micrographs and selected area diffraction patterns (SADPs) were considered, as shown in Figure 4. The annealed coupon did not show any precipitate on the grain boundaries or within the grain, whereas the aged coupon exhibited κ-carbide ((Fe,Mn)_3_AlC) on the grain boundaries and within the grain after aging heat treatment [15,16]. The size of the κ-carbide was several nm, and the κ-carbide was uniformly distributed within the grain. The SADP of the annealing heat-treated coupon did not exhibit any precipitates within the grain (Figure 4b), whereas the SADP of the aged coupon exhibited κ-carbides within the grain and along the grain boundaries (Figure 4d). Fine κ-carbide can prevent dislocation movement during deformation, improving the yield and tensile stresses [5,7].

**Figure 5:**
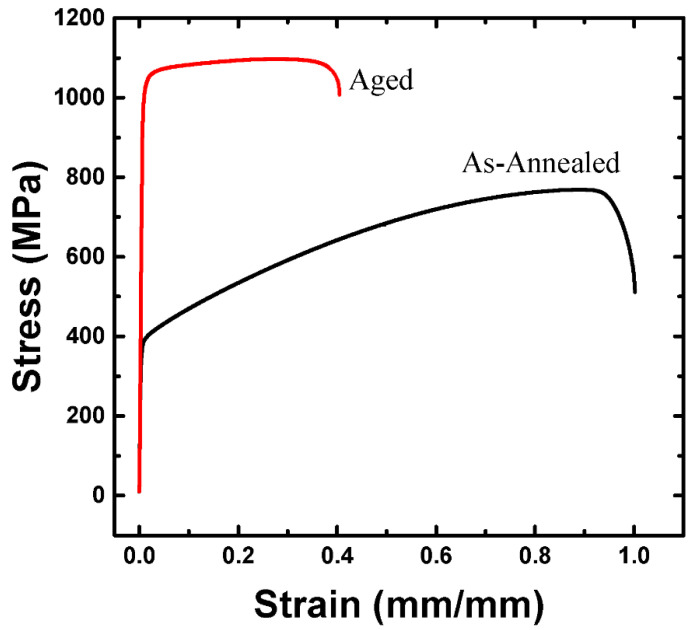
Comparison of the stress-strain curves before and after the aging heat treatment.

Figure 5 shows the engineering stress-strain curves of the specimens obtained from the coupons before and after the aging heat treatment. After aging heat treatment, tensile and 0.2% offset yield stresses were improved by almost two-fold, compared to the stresses before aging heat treatment, whereas the elongation and reduction of the area were considerably reduced. Table 2 shows the tensile and 0.2% offset yield stresses, elongation, and reduction of area, based on the stress-strain curves in Figure 2. The yield ratios of the specimens before and after aging heat treatment were approximately 0.59 and 0.83, respectively. After aging heat treatment, the work-hardening ratio was reduced.

The 0.2% offset yield stress of the aging heat-treated coupon was two-fold higher than that of the annealing heat-treated coupon. This result was attributed to the precipitate of κ-carbide, which effectively prevented the motion of dislocations [17,18]. Work hardening in the aging heat-treated coupon was negligible after the yield point, because the first dislocation on a glide plane sheared the κ-carbide under high resistance and the following dislocation gliding on the same plane faced lower resistance to shear the κ-carbide than the first dislocation [2,17,18].

### 3.3. LCF Properties

**Figure 6:**
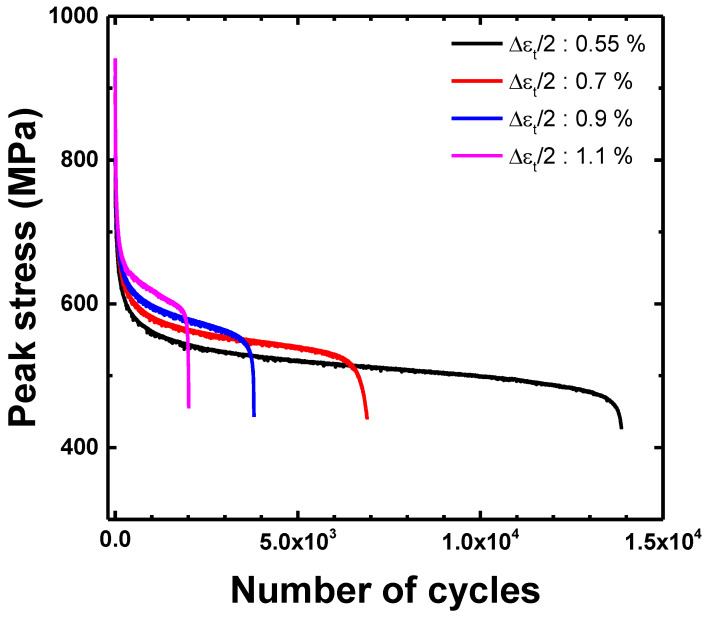
Cyclic stress responses with the total strain amplitude.

**Figure 7:**
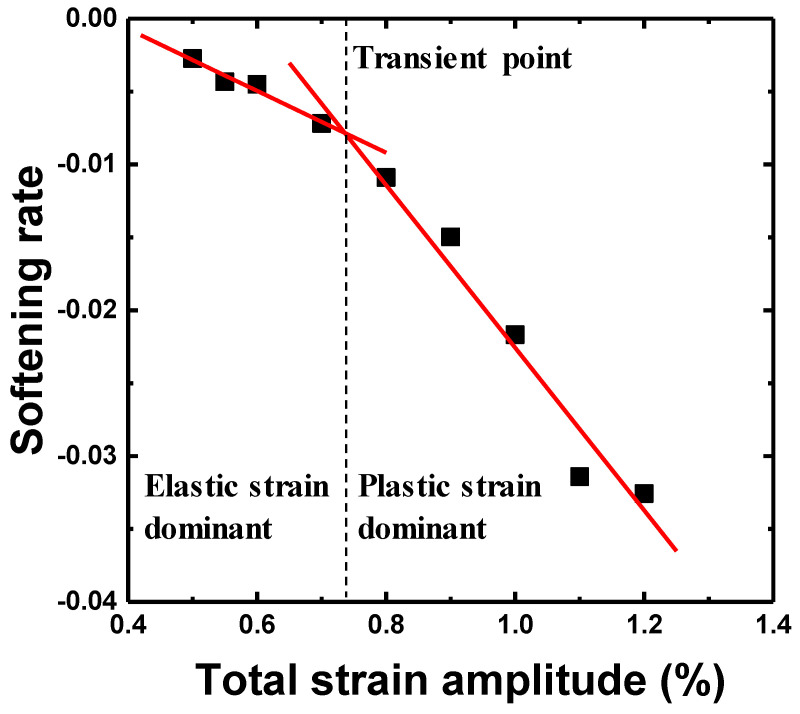
Variation in the cyclic softening rate $\left(d\sigma_{max.}/dN\right)$ at the saturation stage as function of the total strain amplitude.

Figure 6 shows the cyclic maximum stress response under the total strain amplitudes (Δ*ε_t_*/2) of 0.55, 0.7, 0.9, and 1.1%. In all cases, the maximum stress rapidly decreased in the initial stage and showed a saturation stage before final failure. A cyclic softening behavior was observed at all the total strain amplitudes throughout the entire fatigue life. The maximum stress increased with an increase in the total strain amplitude, whereas the LCF life decreased. Figure 7 shows softening rate ($d\sigma_{Max.}/dN$) as a function of the total strain amplitude. The cyclic softening rate was determined by the slope of maximum stress as a function of the cyclic number at the saturation stage, as shown in Figure 6. The softening rate increased with an increase in the total strain amplitude. The slope of the softening rate was changed, based on the transition total strain amplitude (Δ*ε_t_*/2 = 0.73% when Δ*ε_e_*/2 = Δ*ε_p_*/2). The softening rate in the plastic-strain-dominant region was steeper than that in the elastic-strain-dominant region.

**Figure 8:**
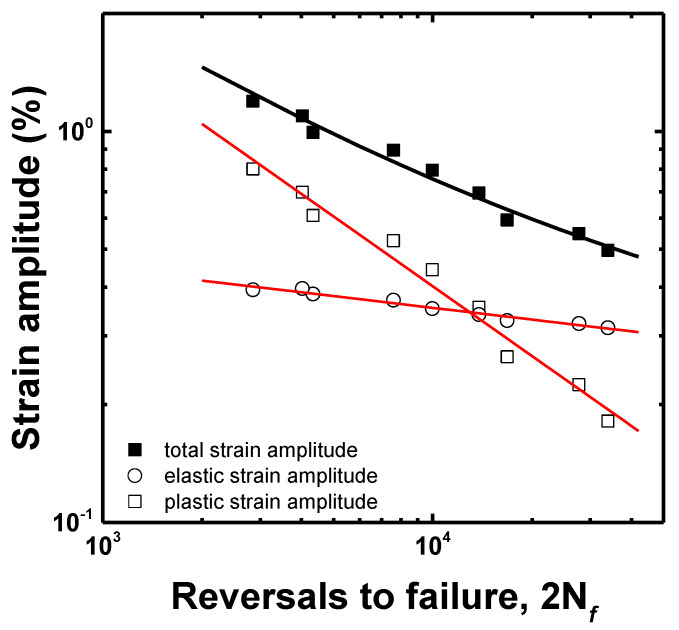
LCF life curve as a function of the number of the reversals to failure.

**Figure 9:**
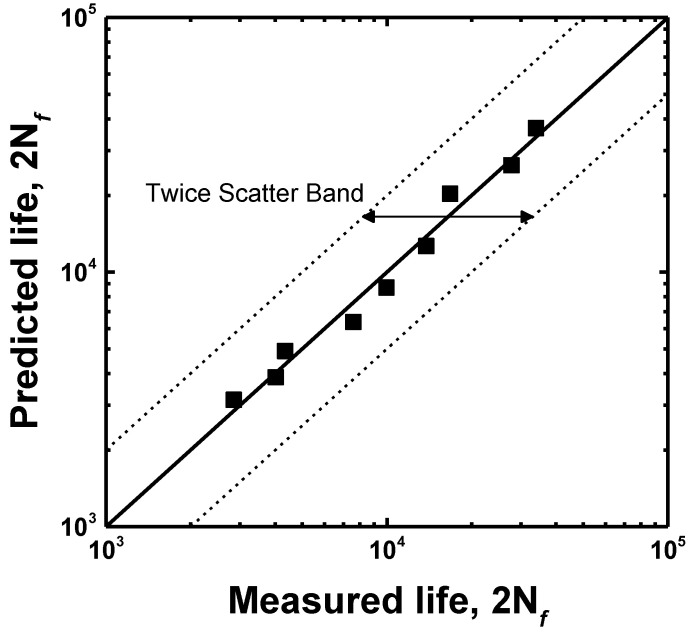
Comparison between the experimental and the predicted LCF life.

The elastic and plastic strain amplitudes were obtained from the elastic and plastic strain components from the hysteresis loop at half the number of cycles to failure. The LCF life followed the Coffin–Manson equation [19,20]:

(1) $$\frac{\Delta \varepsilon_{t}}{2}=\frac{\Delta \varepsilon_{e}}{2}+\frac{\Delta \varepsilon_{p}}{2}=\frac{\sigma_{f}^{\prime }}{E}{\left(2N_{f}\right)}^{b}+\varepsilon_{f}^{\prime }{\left(2N_{f}\right)}^{c}$$

where Δ*ε_t_*/2, Δ*ε_e_*/2 and Δ*ε_p_*/2 are the total, elastic and plastic strain amplitudes, respectively; 2*N_f_*, *σ_f_*, *E*, *ε_f_*, *b*, and *c* are the number of reversals to failure, fatigue strength coefficient, Young’s modulus, fatigue ductility coefficient, fatigue strength exponent, and fatigue ductility exponent, respectively. After the elastic and the plastic strain amplitudes were obtained from the hysteresis loop at the half of the life, the LCF life was calculated, as shown in Figure 8. The LCF life at each total strain amplitude corresponded well with that calculated using the Coffin–Manson equation. The LCF parameters of the Coffin–Manson equation are shown in Table 3. Additionally, the transition point of the softening rate, as shown in Figure 7, corresponded to the transition total strain amplitude crossing the elastic and plastic strain amplitudes. From this result, it was estimated that the plastic-strain-amplitude-dominant region increased the softening rate and reduced the LCF life. To estimate the accuracy of the LCF life, as calculated using Equation (1), the result was compared to the experimental life, as shown in Figure 9, which confirmed that all the LCF data points were distributed within the “twice scatter band”, which is the correspondence confidence interval between the experimental and the predicted results. Therefore, it was confirmed that the LCF behavior of the lightweight steel could be well described by the Coffin–Manson equation.

**Figure 10:**
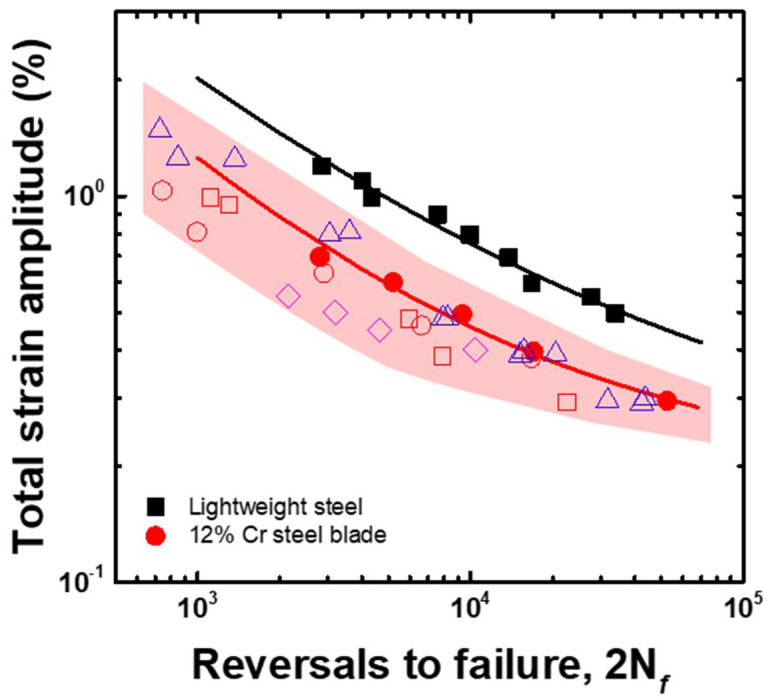
Comparison of the LCF life of 12% Cr and lightweight steels. Solid spots represent experimental results and open spots are from references. open squares and open circles [14], open triangles [15], and open diamonds [16].

To estimate the applicability of the lightweight steel for steam turbine blades, its LCF life was compared with that of the 12% Cr steel, as shown in Figure 10. The commercial 12% Cr steel sample exhibited an LCF life that was very similar to those reported by previous studies [21,22,23], whereas the LCF life of lightweight steel was three times that of the commercial 12% Cr steel over the total strain amplitude range of 0.5 to 1.2%. Therefore, the lightweight steel has a high applicability for steam turbine blades, as the design life for steam turbine blades is determined by the LCF life.

**Figure 11:**
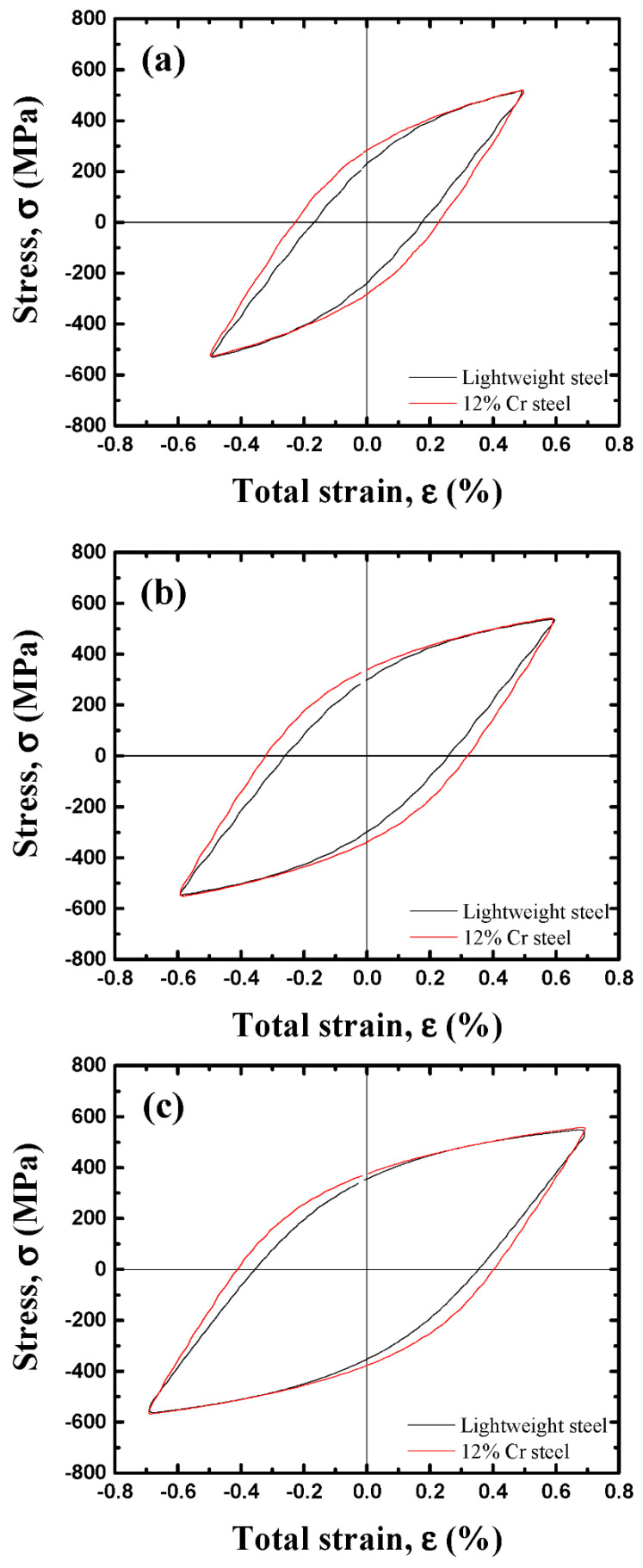
Comparison of the stress-strain hysteresis loops between the lightweight and 12% Cr steels for the half-life cycle at the total strain amplitudes of (**a**) 0.5, (**b**) 0.6, and (**c**) 0.7%.

To understand why lightweight steel has a longer LCF life than that of 12% Cr steel, stress-strain hysteresis loops and the deformation microstructure were analyzed and discussed. The typical hysteresis loops recorded during the LCF test for the half-life cycle were compared at the total strain amplitudes of 0.5, 0.6, and 0.7%, as shown in Figure 11. In both steels, as the total strain amplitude increased from 0.5 to 0.7%, the plastic-strain-amplitude component and the maximum stress value tended to increase. The plastic strain component of 12% Cr steel at the three different total strain amplitudes was larger than that of the lightweight steel. It is well known that at identical total strain amplitudes, a lower plastic component of a material increases its LCF life [24,25]. Therefore, the lightweight steel exhibited a longer LCF life than that of the commercial 12% Cr steel.

**Figure 12:**
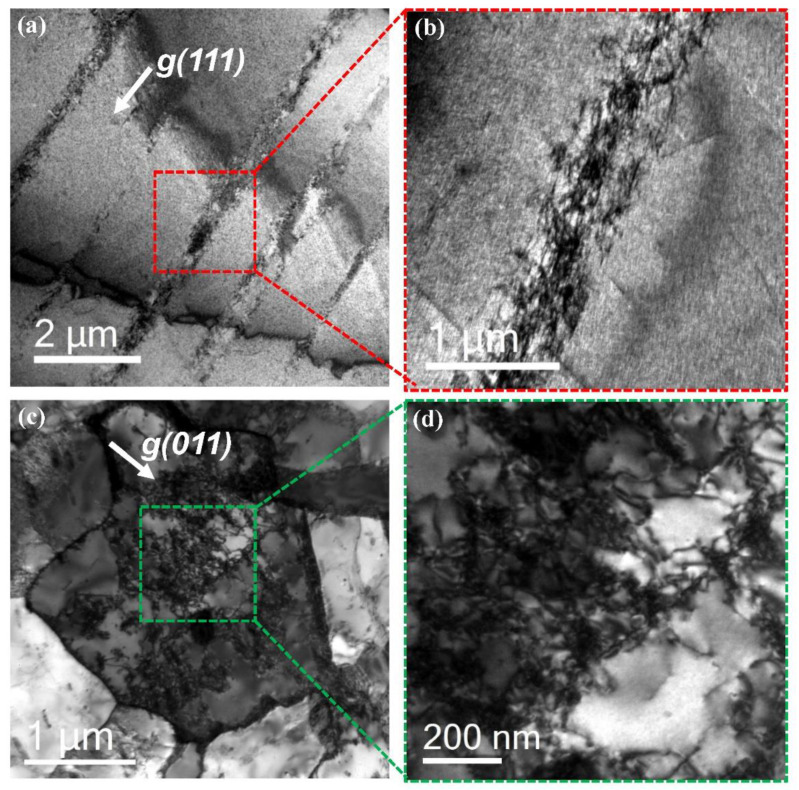
(**a**,**b**) Bright-field (BF) TEM images of the deformed lightweight steel and (**c**,**d**) BF TEM images of the deformed 12% Cr steel.

The deformation structure after the LCF test was analyzed using TEM to understand the reason for the improved LCF life. Figure 12 shows the dislocation structure of the lightweight (Figure 12a,b) and 12% Cr steels (Figure 12c,d) after the LCF test. The dislocation structure of the lightweight steel shows that the generation and movement of dislocation during deformation occurred intensively on the confined single-slip planes. It is well known that the localized planar dislocation glide in the lightweight steel is attributed to the κ-carbide precipitated within grain [3,12,22]. That is, after the first dislocation on a glide plane shears the κ-carbide, the shearing of κ-carbide by the following dislocations on the same glide plane can occur more easily; this is called “glide plane softening”. The planar-dislocation-glide phenomenon prevents entanglement by the interaction among dislocations due to the arrangement effects of the dislocation movement. Thus, this behavior avoids the effects of localized stress concentration due to dislocation entanglement and results in a delayed effect of strain hardening [22]. However, the dislocations in the 12% Cr steel show an entangled structure typically observed in the deformation structure of a tempered martensite structure. The steel exhibited a deformation dislocation structure with a typical dislocation cell structure, as the generation and movement of dislocations occurred simultaneously on different {110} planes during deformation in martensitic steel. When such entanglements among the dislocations are activated, the localized stress concentration is induced, increasing the probability of crack initiation. In summary, it is thought that the failure delay in lightweight steel may be induced by the minimized entanglement among the dislocations due to the characteristic planar dislocation glide during the fatigue strain.

## 4. Conclusions

In this study, a large Fe-Mn-Al-C-based lightweight steel with a fine and uniform austenitic structure was successfully manufactured, using steelmaking, forging, and rolling processes in a production facility. The variation in properties and the LCF life of the lightweight steel were evaluated. The following conclusions were obtained:

1. After the rolling process, the Fe-Mn-Al-C-based lightweight steel that was successfully manufactured as a 1.5-ton class plate showed a fine and uniform austenitic structure. After annealing and aging heat treatments, κ-carbides with a size of several nm were uniformly precipitated within the grain and on the grain boundaries.
2. The aging heat-treated lightweight steel exhibited a cyclic softening behavior during the LCF test and the LCF life was predicted using the Coffin–Manson equation.
3. The LCF life of the lightweight steel over the total strain amplitude range of 0.5–1.2% was three times longer than that of the commercial 12% Cr steel that is currently used in steam turbines.
4. The localized planar dislocation glide by the κ-carbide minimized the entanglement among dislocations, and this characteristic dislocation movement extended the LCF life of the lightweight steel.

## Figures and Tables

**Table 1:** Appearance and dimensions of the as-rolled lightweight steel; the locations of the microstructural samples were determined.

Composition	C	Si	Mn	P	S	Al
wt.%	0.90	0.28	29.0	0.026	0.001	7.9

**Table 2:** Tensile properties of the as-annealed and the aged lightweight steels.

	0.2% Y.S. (MPa)	T.S. (MPa)	El. (%)	R.A. (%)
As-annealed	454	768.8	89	75
Aged	850	1027.3	27	44

**Table 3:** LCF parameters of the aged lightweight steel.

Parameters	$\sigma_{f}^{\prime }/E$	*b*	$\varepsilon_{f}^{\prime }$	*C*
Aged	0.8859	−0.0997	94.9139	−0.5934

## Data Availability

The data presented in this study are available on request from the corresponding auther or the first auther.

## References

[1] Frommeyer G., Brüx U. (2006). Microstructures and mechanical properties of high-strength Fe-Mn-Al-C light-weight TRIPLEX steels. Steel Res. Int..

[2] Choi K., Seo C.H., Lee H., Kim S.K., Kwak J.H., Chin K.G., Park K.T., Kim N.J. (2010). Effect of aging on the microstructure and deformation behavior of austenite base lightweight Fe-28Mn-9Al-0.8C steel. Scripta Mater..

[3] Gutierrez-Urrutia I., Raabe D. (2013). Influence of Al content and precipitation state on the mechanical behavior of austenitic high-Mn low-density steels. Scripta Mater..

[4] Sutou Y., Kamiya N., Umino R., Ohnuma L., Ishida K. (2010). High-strength Fe-20Mn-Al-C-based alloys with low density. ISIJ Int..

[5] Park J.Y., Park S., Lee J., Moon J., Lee T.H., Jeong K.J., Han H.N., Shin J. (2017). Effect of cooling rate on the microstructure and mechanical properties of Fe-M-Al-C light-weight steels. Korean J. Met. Mater..

[6] Lee K., Park S., Lee J., Moon J., Kang J., Kim D., Suh J., Han H.N. (2016). Effect of aging treatment on microstructure and intrinsic mechanical behavior of Fe-31.4Mn-11.4Al-0.89C lightweight steel. J. Alloy Compd..

[7] Acselrad O., Kalashnikov I.S., Silva E.M., Khadyev M.S., Simao R.A. (2006). Diagram of phase transformation in the austenite of hardened alloy Fe-28%Mn-8.5%Al-1%C-1.25%Si as a result of aging due to isothermal heating. Met. Sci. Heat Treat..

[8] Mukhopadhyay N.K., Chowdhury S.G., Das G., Chattoraj I., Das S.K., Bhattacharya D.K. (1998). An investigation of the failure of low pressure steam turbine blades. Eng. Fail. Anal..

[9] Booysen C., Heyns P.S., Hindley M.P., Scheepers R. (2015). Fatigue life assessment of a low pressure steam turbine blade during transient resonant conditions using a probabilistic approach. Int. J. Fatigue.

[10] Saxena A. (1998). Nonlinear Fracture Mechanics for Engineers.

[11] Moon J., Park S.J., Lee C., Han H.N., Lee T.H., Lee C.H. (2017). Microstructure evolution and age-hardening behavior of microalloyed austenitic Fe-30Mn-9Al-0.9C light-weight steel. Met. Mater. Trans. A.

[12] Haase C., Zehnder C., Ingendahl T., Bikar A., Tang F., Hallstedt B., Hu W., Bleck W., Monodove D.A. (2017). On the deformation behavior of κ-carbide-free and κ-carbide-containing high-Mn light-weight steel. Acta Mater..

[13] Kim C.W., Kwon S.I., Lee B.H., Moon J.O., Park S.J., Lee J.H., Hong H.U. (2016). Atomistic study of nano-sized κ-carbide formation and its interaction with dislocationsin a cast Si added FeMnAlC lightweight steel. Mater. Sci. Eng. A.

[14] Yan M., Li Y., Li H., Sun Y., Qin H., Zheng Q. (2019). Preparation and ladle slag resistance mechanism of MgAlON bonded Al_2_O_3_-MgAlON-Zr_2_Al_3_C_4_-(Al2Co)_1_-x(AlN)x refractories. Ceram. Int..

[15] Shin J., Rim G., Kim S., Jang J.H., Park S., Lee J. (2020). Effects of aging heat-treatment on dynamic strain aging behavior in high-Mn lightweight steel. Mater. Charact..

[16] Shin J., Jang J.H., Kim S., Park S., Lee J. (2020). Dynamic strain aging in Fe-Mn-Al-C lightweight steel. Phil. Mag. Lett..

[17] Wang Z., Lu W., Zhao H., He J., Wang K., Zhou B., Ponge D., Raabe D., Li Z. (2020). Formation mechanism of κ-carbides and deformation behavior in Si-alloyed FeMAlC lightweight steels. Acta Mater..

[18] Yao M.J., Welsch E., Ponge D., Haghighat S.M.H., Sandlöbes S., Choi P., Herbig M., Bleskov I., Hickel T., Lipinska-Chwalek M. (2017). Strengthening and strain hardening mechanisms in a precipitation-hardened high-Mn lightweight steel. Acta. Mater..

[19] Dieter G.E. (1988). Mechanical Metallurgy.

[20] Shin J., Kim Y., Lee J. (2018). Effects of grain size on the fatigue properties in cold-expanded austenitic HNSs. Met. Mater. Int..

[21] Kanazawa K., Yamaguchi K., Kobayashi K. (1979). The temperature dependence of low cycle fatigue behavior of martensitic stainless steels. Mater. Sci. Eng. A.

[22] Degallaix G., Vogt J.B., Foct J. (1987). Low cycle fatigue of a 12% Cr martensitic stainless steel: The role of microstructure. Low Cycle Fatigue and Elasto-Plastic Behavior of Materials.

[23] Jeong I.H., Park Y.M., Bae M.K., Kim C.H., Kim T.G. (2019). A study on low-cycle fatigue of high Chromium heat-resistant steel. Mod. Phys. Lett. B.

[24] Lefebvre D., Ellyin F. (1984). Cyclic response and inelastic strain energy in low cycle fatigue. Int. J. Fatigue.

[25] Mishnev R., Dudova N., Kaibyshev R. (2017). Effect of the strain rate on the low cycle fatigue behavior of a 10Cr-2W-Mo-3CO-NbV steel at 650 °C. Int. J. Fatigue.

